# Associations of Socioeconomic Status, Public vs Private Insurance, and Race/Ethnicity With Metastatic Sarcoma at Diagnosis

**DOI:** 10.1001/jamanetworkopen.2020.11087

**Published:** 2020-08-07

**Authors:** Brandon J. Diessner, Brenda J. Weigel, Paari Murugan, Lin Zhang, Jenny N. Poynter, Logan G. Spector

**Affiliations:** 1 Division of Epidemiology and Clinical Research, Department of Pediatrics, University of Minnesota, Minneapolis; 2 Division of Pediatric Hematology/Oncology, Department of Pediatrics, University of Minnesota, Minneapolis; 3 Department of Laboratory Medicine and Pathology, University of Minnesota, Minneapolis; 4 Division of Biostatistics, University of Minnesota, Minneapolis

## Abstract

**Importance:**

Approximately 10% to 30% of patients with sarcoma present with detectable metastases at diagnosis. However, the extent to which presentation with metastases is due to delayed diagnosis vs other factors remains unclear.

**Objective:**

To evaluate whether socioeconomic status, insurance status, or race/ethnicity were associated with the presence of metastases at diagnosis of sarcoma.

**Design, Setting, and Participants:**

This cross-sectional study used data from the population-based Surveillance, Epidemiology, and End Results program. Adult and pediatric patients with an initial diagnosis of soft-tissue and bone sarcoma between 2001 and 2015 were stratified by age group (pediatric, <20 years; adult, 20-65 years; older adult, >65 years) and sarcoma subtype. Statistical analyses were performed between August 2019 and January 2020.

**Exposures:**

Surveillance, Epidemiology, and End Results Census tract–level socioeconomic status index, insurance status, and race/ethnicity.

**Main Outcomes and Measures:**

The odds of presenting with metastases at diagnosis were calculated.

**Results:**

A total of 47 337 patients with first primary malignant sarcoma were included (24 343 male patients [51.4%]), with 29 975 non-Hispanic White patients (63.3%), 5673 non-Hispanic Black patients (12.0%), 7504 Hispanic patients (15.8%), and 4185 American Indian–Alaskan Native and Asian Pacific Islander patients (8.8%). Liposarcoma in adults was the only subtype and age group combination that demonstrated a significant trend in incidence across socioeconomic status levels (odds ratio, 0.85; 99% CI, 0.76-0.96; *P* = .001). However, compared with having non-Medicaid insurance, having Medicaid or no insurance in adults was associated with an increased odds of metastases at diagnosis for 6 of the 8 sarcoma subtypes evaluated; osteosarcoma and Ewing sarcoma were the only 2 subtypes in adults for which metastases were not associated with insurance status. In addition, there was an increased risk of presenting with metastases among non-Hispanic Black adults diagnosed with leiomyosarcoma (odds ratio, 1.87; 99% CI, 1.41-2.48) and unclassified sarcomas (odds ratio, 1.65; 99% CI, 1.01-2.67) compared with non-Hispanic White adults that was independent of socioeconomic and insurance status.

**Conclusions and Relevance:**

These findings suggest that delayed access to care is associated with advanced stage at diagnosis for several soft-tissue sarcoma subtypes in adults, whereas other factors may be associated with the metastatic progression of osteosarcoma and Ewing sarcoma, as well as the racial disparities observed with metastatic leiomyosarcoma and unclassified sarcomas.

## Key Points

**Question:**

Is the presence of metastases at diagnosis of sarcoma associated with socioeconomic status, race/ethnicity, or insurance status?

**Findings:**

In this cross-sectional, population-based study of 47 337 sarcoma cases, metastases at diagnosis of sarcoma was not associated with socioeconomic status as measured by small-area Census characteristics. Among adults, those with Medicaid insurance or no insurance had a higher odds of metastases at diagnosis of most soft-tissue sarcomas, but not bone sarcomas, whereas the racial disparities in the prevalence of metastatic leiomyosarcoma and unclassified sarcomas were not associated with small-area socioeconomic status and insurance status.

**Meaning:**

These findings suggest that Medicaid insurance or no insurance is associated with the presence of metastases at the time of diagnosis among adults with soft-tissue sarcomas, suggesting a diagnostic delay, but there is no such association for adults with bone sarcomas.

## Teaser

This cross-sectional study evaluates whether socioeconomic status, insurance status, or race/ethnicity are associated with the presence of metastases at the time of diagnosis of sarcoma.

## Introduction

Soft-tissue sarcomas (STSs) and bone sarcomas comprise a group of more than 50 histologically distinct and exceedingly rare malignant tumors.^1^ Approximately 10% to 30% of patients with sarcoma present with detectable metastases at initial diagnosis, depending on subtype.^2,3,4^ Those with metastatic disease at diagnosis experience poorer outcomes than those with localized disease, with overall 5-year survival rates ranging from 10% to 30% for those with metastases and 65% to 80% for those without metastases.^5,6,7,8,9^ Sarcomas cause few early recognizable signs and symptoms that can often lead to an extended delay between symptom onset and a definitive diagnosis.^10,11^ However, the extent to which a delayed diagnosis increases the likelihood of metastases and a higher risk of death from sarcoma remains unclear.^10,11,12,13^

A lower socioeconomic status (SES) may be representative of factors at the individual or community level that prevent patients from receiving timely access to medical care^14,15^ and has been associated with advanced stage at diagnosis of more common cancers, including those of the breast, prostate, colorectal, lung, and cervix.^16^ Prior analyses^2,3^ of the effect of SES on the diagnosis of sarcoma in the US are few and largely based on county-level analyses of SES, which may have introduced bias, considering that counties may comprise economically heterogeneous populations. Further insight can be obtained from analyses of race/ethnicity because any observed associations that are independent of SES may point toward variation in genetic risk for presentation with metastases. Results from analyses of race/ethnicity are limited by the extreme rarity of the disease, often constraining investigators to analyze histologically distinct sarcoma subtypes as a single group (eg, sarcoma) and few adjusted for likely confounding by SES.^17,18^ Of note, a genome-wide association study^19^ of osteosarcoma identified 2 germline genetic variants that were associated with an increased risk of metastases. This indicates that the metastatic potential for at least 1 sarcoma subtype is present at the start of tumorigenesis and not solely through sequential accumulation of mutations during disease progression.

The purpose of this cross-sectional study was to use a large, contemporary cohort of patients with sarcoma within the Surveillance, Epidemiology, and End Results (SEER) registry to describe associations of small-area SES, race/ethnicity, and insurance status with metastases present at the diagnosis of sarcomas across the age spectrum. The results of this study provide a comprehensive overview regarding these associations among individually rare subtypes of sarcoma.

## Methods

### Study Population

We used the specialized SEER Census Tract–level SES Database,^20^ which includes cancer cases diagnosed between 2000 and 2015 in the catchment area of 16 SEER registries; the Alaska Native and Louisiana Tumor registries are excluded.^21^ Microscopically confirmed, first primary malignant sarcoma cases were identified using *International Classification of Disease for Oncology, 3rd Edition* (*ICD-O-3*) histology codes recognized by the 2013 World Health Organization’s *Classification of Tumours of Soft Tissue and Bone*.^22^ We then categorized sarcoma cases into subtypes of interest according to the World Health Organization classifications and the recommendations of an expert sarcoma oncologist (B.J.W.) and pathologist (P.M.) (eTable 1 in the Supplement). Kaposi sarcoma was excluded from analyses because it primarily arises in the setting of HIV infection.^23^ We also excluded cases diagnosed in the year 2000 because they were not directly coded under the *ICD-O-3* histology coding guidelines introduced in 2001.^24^

This study is exempt from institutional review board approval and the need for informed consent because it involves the use of publicly available, deidentified data in accordance with 45 CFR §46. This study follows the Strengthening the Reporting of Observational Studies in Epidemiology (STROBE) reporting guideline for cross-sectional studies.^25^

### Metastatic Disease at Diagnosis, SES, Race/Ethnicity, and Other Covariate Categories

Metastatic sarcoma was characterized on the basis of the SEER summary staging variable. Cases with a staging of *distant* were classified as having metastases at diagnosis, whereas those with a staging of *localized* or *regional* were not. Cases with an unknown stage at diagnosis were excluded from further analyses. Race and ethnicity were evaluated using mutually exclusive groups—non-Hispanic White, non-Hispanic Black, Asian Pacific Islander (API) or American Indian and Alaska Native (AIAN), and Hispanic—that were assigned to an individual according to the reported race/ethnicity in a patient’s medical records. The identification of individuals of Hispanic ethnicity was further enhanced by use of the North American Association for Central Cancer Registries Hispanic-Latino identification algorithm, which is based principally on surname and birth place.^26^ We combined the API and AIAN race categories because of sample size constraints. Five-year age groups were categorized into 9 broader age group categories to account for small sample sizes: 0 to 9 years, 10 to 19 years, 20 to 29 years, 30 to 39 years, 40 to 49 years, 50 to 64 years, 65 to 74 years, 75 to 84 years, and 85 years and older.

Small-area SES was analyzed from a composite index. The index is calculated by SEER using a principal component analysis of several Census tract–level SES indicator variables as specified by Yost et al^27^: median household income, median house value, median rent, percentage of the population below 150% of the poverty line, an education index, percentage of the population with working class occupations, and percentage of the population older than 16 years in the workforce without a job. Cases were geocoded to a Census tract according to their address at diagnosis. The SES indices were linked to a Census tract using either the 2000 Decennial Census long-form survey or a series of American Community Survey 5-year estimates, depending on year of diagnosis. The SES index is provided to SEER users as a 5-level categorical variable, with the first category representing the lowest SES quintile. Cases with a missing SES index or an address that could not be geocoded to a Census tract were excluded from analysis.

### Statistical Analyses

**Figure 1.**
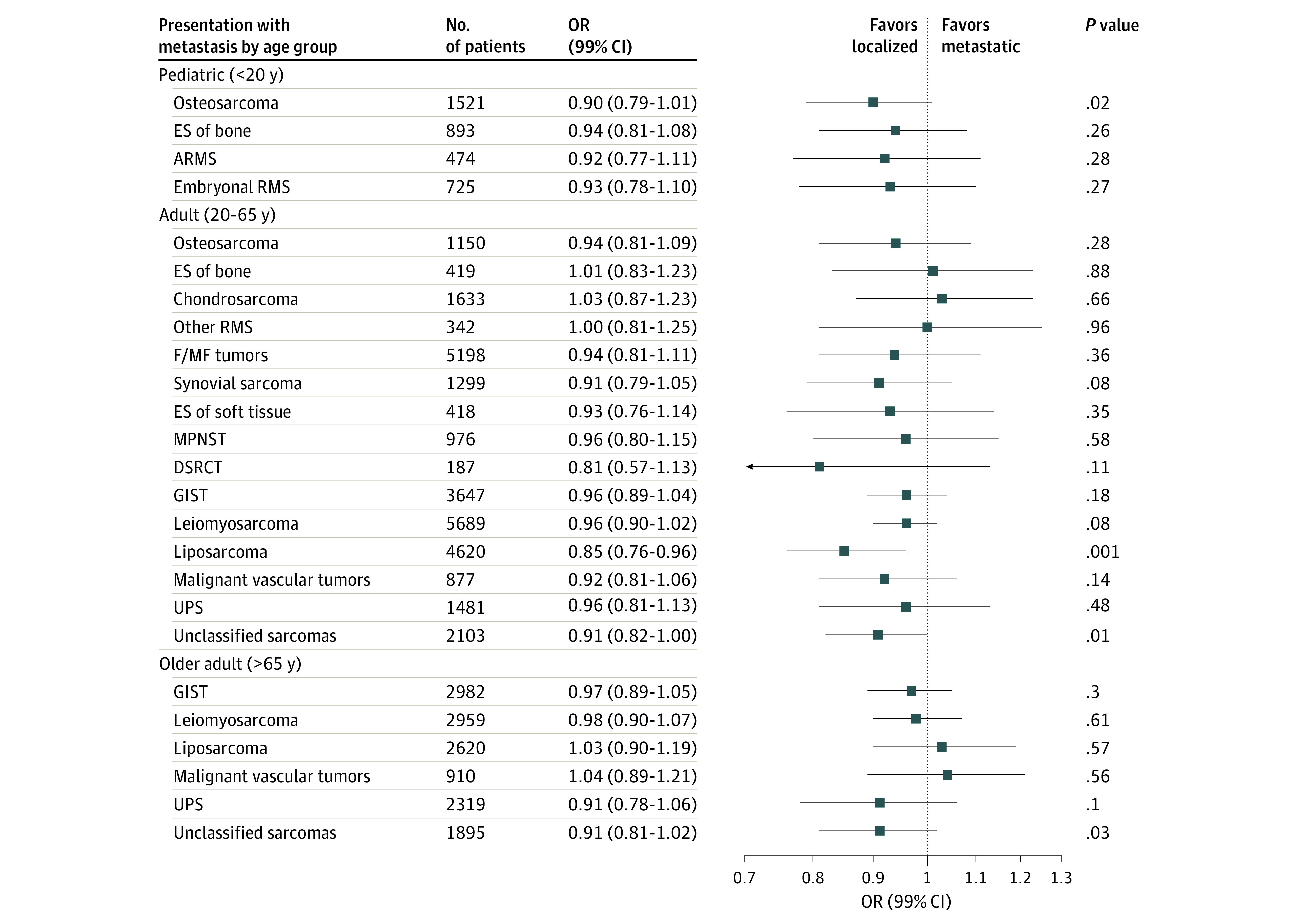
Multivariable Adjusted Odds Ratios (ORs) and 99% CIs for Metastatic Sarcoma by Socioeconomic Status, Stratified by Age Group and Sarcoma Subtype: Surveillance, Epidemiology, and End Results 16 Registries, 2001-2015 All data are adjusted for race, sex, age at diagnosis, and year of diagnosis. ARMS indicates alveolar rhabdomyosarcoma; DSRCT, desmoplastic small round cell tumor; ES, Ewing sarcoma; F/MF, fibroblastic or myofibroblastic; GIST, gastrointestinal stromal tumor; MPNST, malignant peripheral nerve sheath tumor; RMS, rhabdomyosarcoma; and UPS, undifferentiated pleomorphic sarcoma.

Multivariable logistic regression models were used to evaluate the odds of presenting with metastases at diagnosis across quintiles of small-area SES (hereafter referred to as SES) and race/ethnicity, adjusted for age at diagnosis, sex, and year of diagnosis. Covariates were selected a priori for inclusion on the basis of previous associations with sarcoma and/or our exposures of interest.^9,28,29^ All models were stratified by broad age group categories (pediatric, <20 years; adult, 20-65 years; and older adult, >65 years). Sixty-five years was chosen as the cutoff point for older adults because it is the age of Medicare eligibility. Within each age group strata, associations were reported for any sarcoma subtype with more than 100 metastatic cases, a cutoff that was chosen a priori to maintain adequate power in strata specific analyses. For all sarcomas, SES was assessed as either a categorical variable (with the lowest quintile serving as the reference) or an ordinal variable, with the *P* value from the ordinal variable serving as a test for trend. We focus our reporting here on results from SES evaluated as an ordinal variable (Figure 1); results from SES evaluated as a categorical variable are presented in eTable 2 in the Supplement.

In addition, we conducted multivariable logistic regression analyses to evaluate the odds of metastases at diagnosis by insurance status, while controlling for small-area SES (as an ordinal variable), race/ethnicity, age at diagnosis, sex, and year of diagnosis. Insurance status was defined according to the SEER variable as non-Medicaid insurance (insured or insured and no specifics), Medicaid coverage (any Medicaid), or uninsured. Analyses were confined to sarcoma subtypes with more than 100 metastatic cases diagnosed after 2007, when insurance status data began to be collected by SEER. We further restricted analyses to cases diagnosed in the adult age group strata because few pediatric or older adult cases were uninsured. Cases with unknown insurance status were excluded from analysis.

A *P* < .01 was considered statistically significant. This significance threshold was used because it is approximately equal to 0.05 divided by 4, the number of hypothesis tests conducted within each sarcoma subtype and age group (ie, the ordinal SES and 3 race/ethnicity comparisons). All reported *P* values are 2-sided and are calculated from logistic regression models that used the Wald χ^2^ statistic. All data sets were generated using SEER*stat statistical software version 8.3.6 (National Cancer Institute),^30^ and statistical analyses were performed in R statistical software version 3.4.4 (R Project for Statistical Computing).^31^ Statistical analyses were performed between August 2019 and January 2020.

## Results

eTable 3 in the Supplement presents the clinical and demographic distribution of included cases. After excluding 3613 cases with unknown stage of diagnosis, 836 cases with a missing SES index, and 705 cases with missing race/ethnicity, we analyzed 90.7% of sarcoma cases with a first primary, malignant sarcoma subtype of interest diagnosed between 2001 and 2015 in the SEER 16 registries (47 337 of 52 183 cases). There was a total of 24 343 male patients (51.4%) and 22 994 female patients (48.6%). With regard to race/ethnicity, 29 975 patients (63.3%) were non-Hispanic White, 5673 (12.0%) were non-Hispanic Black, 7504 (15.8%) were Hispanic, and 4185 (8.8%) were AIAN or API. Most patients with sarcoma (30 039 patients [63.5%]) were aged 20 to 65 years at diagnosis. Overall, the proportion of patients presenting with metastatic disease at diagnosis was 17.3% for STS and 20.5% for bone sarcomas. Among the subtypes evaluated, the prevalence of metastases at diagnosis was highest for alveolar rhabdomyosarcoma (223 patients [47.0%]) among pediatric patients, desmoplastic round cell tumor (134 patients [71.7%]) among adult patients, and gastrointestinal stromal tumors (698 patients [23.4%]) among older adult patients.

### Socioeconomic Status and Metastasis at Diagnosis

Figure 1 shows the odds ratios (ORs) and 99% CIs for presenting with metastatic disease at diagnosis by SES, stratified by sarcoma category and age group strata (crude results are presented in eTable 4 in the Supplement). Liposarcoma in adults was the only subtype and age group combination evaluated to show a significant association, with 15% lower odds of metastatic liposarcoma observed for every increase in SES quintile (OR, 0.85; 99% CI, 0.76-0.96; *P* = .001).

### Race/Ethnicity and Metastasis at Diagnosis

**Figure 2.**
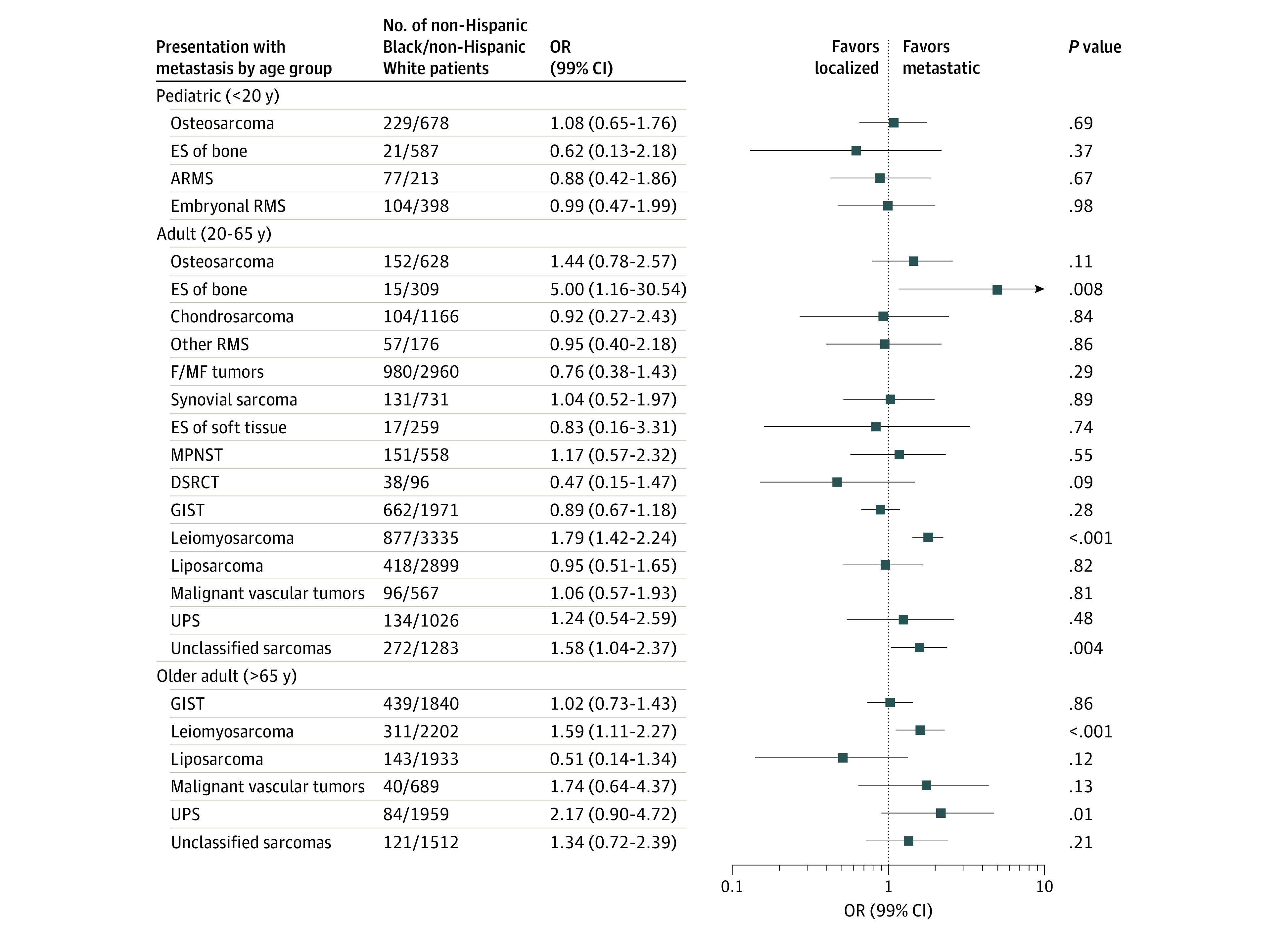
Multivariable Adjusted Odds Ratios (ORs) and 99% CIs for Metastatic Sarcoma in non-Hispanic Black vs non-Hispanic White Patients, Stratified by Age Group and Sarcoma Subtype: Surveillance, Epidemiology, and End Results 16 Registries, 2001-2015 All data are adjusted for small-area socioeconomic status (ordinal variable), sex, age at diagnosis, and year of diagnosis. ARMS indicates alveolar rhabdomyosarcoma; DSRCT, desmoplastic small round cell tumor; ES, Ewing sarcoma; F/MF, fibroblastic or myofibroblastic; GIST, gastrointestinal stromal tumor; MPNST, malignant peripheral nerve sheath tumor; RMS, rhabdomyosarcoma; and UPS, undifferentiated pleomorphic sarcoma.

**Figure 3.**
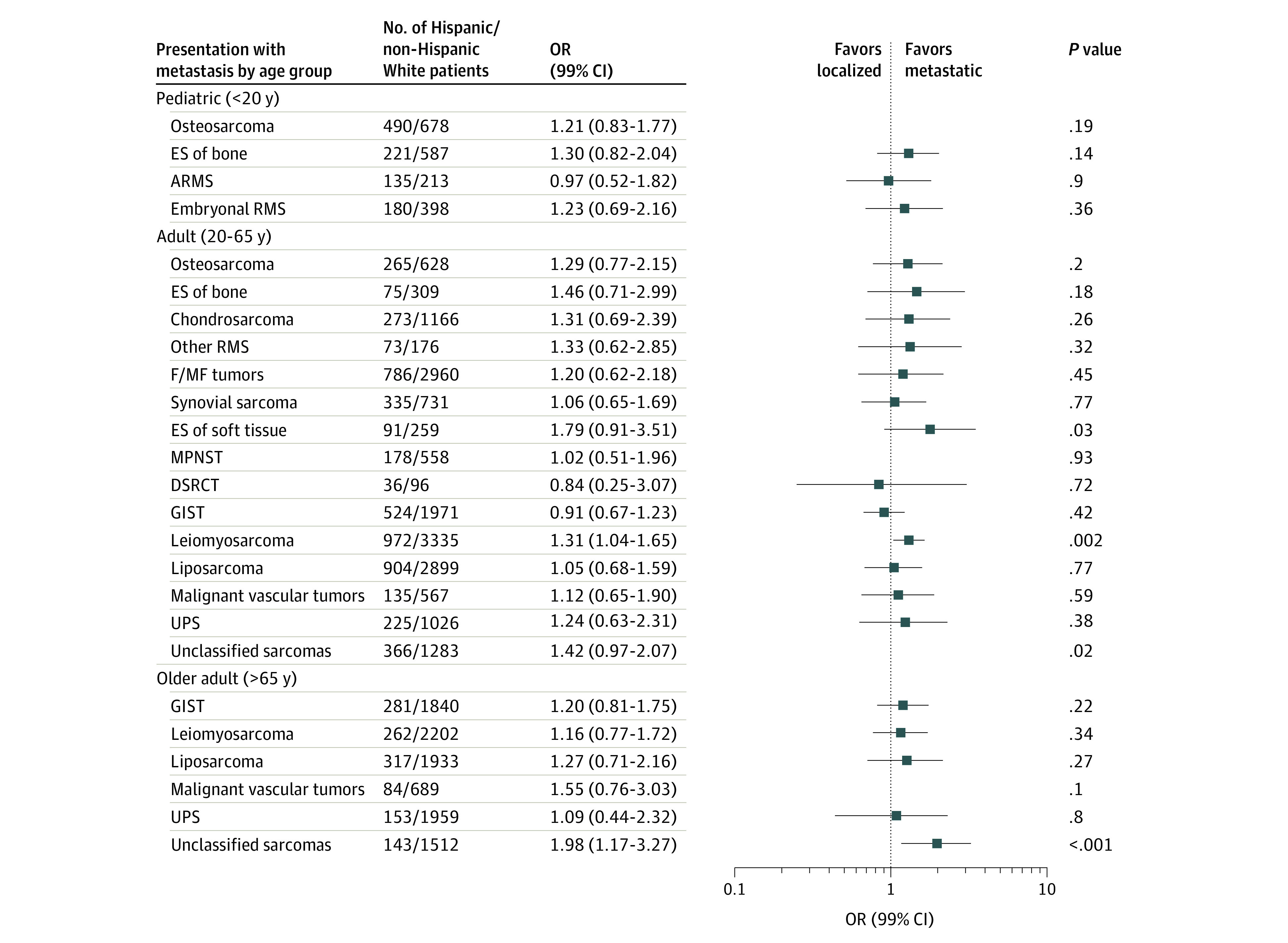
Multivariable Adjusted Odds Ratios (ORs) and 99% CIs for Metastatic Sarcoma in Hispanic vs non-Hispanic White Patients, Stratified by Age Group and Sarcoma Subtype: Surveillance, Epidemiology, and End Results 16 Registries, 2001-2015 ARMS indicates alveolar rhabdomyosarcoma; DSRCT, desmoplastic small round cell tumor; ES, Ewing sarcoma; F/MF, fibroblastic or myofibroblastic; GIST, gastrointestinal stromal tumor; MPNST, malignant peripheral nerve sheath tumor; RMS, rhabdomyosarcoma; and UPS, undifferentiated pleomorphic sarcoma.

**Figure 4.**
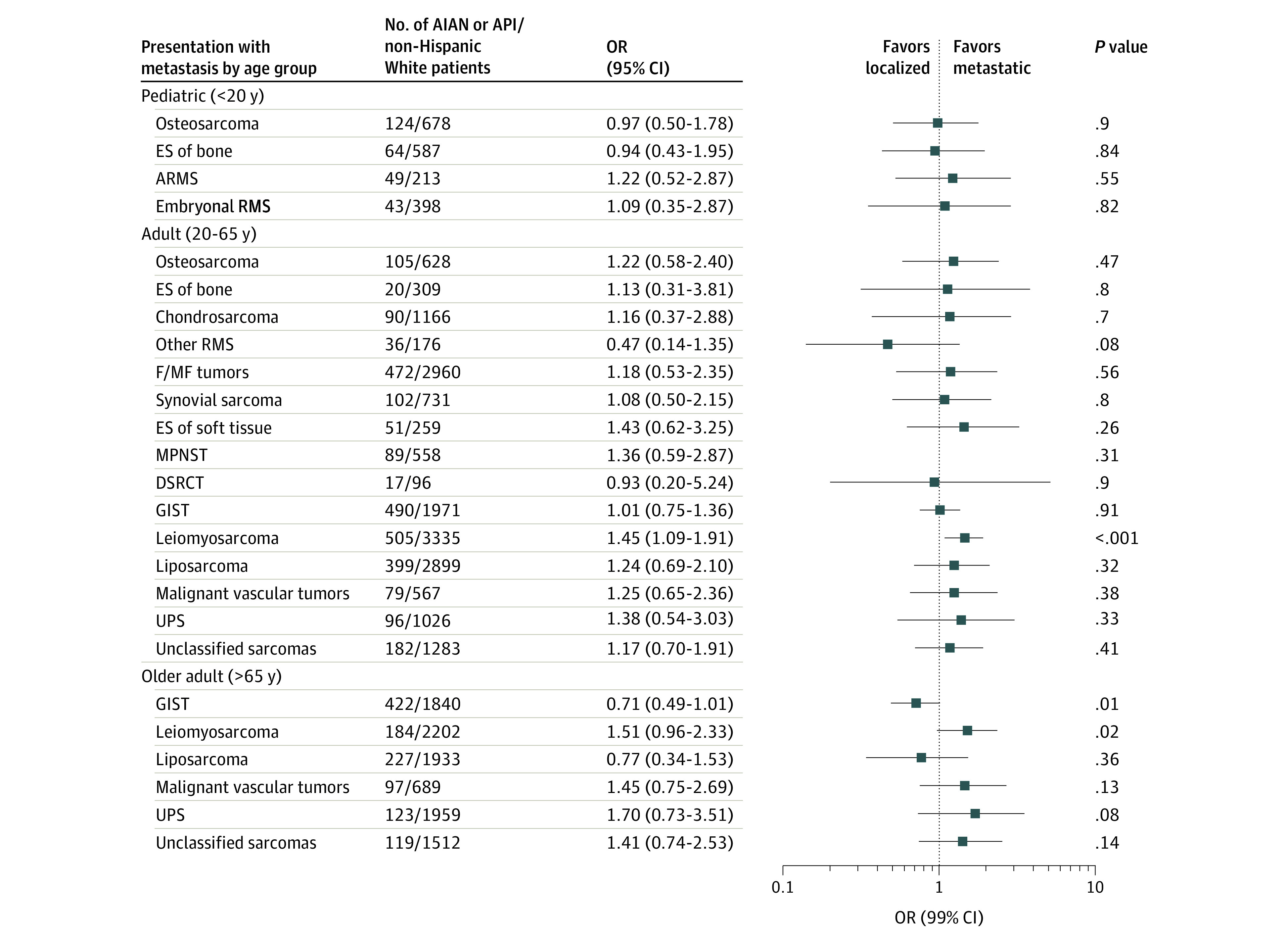
Multivariable Adjusted Odds Ratios (ORs) for Metastatic Sarcoma in American Indian and Alaska Native (AIAN) or Asian Pacific Islander (API) Patients vs non-Hispanic White Patients, Stratified by Age Group and Sarcoma Subtype: Surveillance, Epidemiology, and End Results 16 Registries, 2001-2015 ARMS indicates alveolar rhabdomyosarcoma; DSRCT, desmoplastic small round cell tumor; ES, Ewing sarcoma; F/MF, fibroblastic or myofibroblastic; GIST, gastrointestinal stromal tumor; MPNST, malignant peripheral nerve sheath tumor; RMS, rhabdomyosarcoma; and UPS, undifferentiated pleomorphic sarcoma.

Figure 2, Figure 3, and Figure 4 show the adjusted ORs and 99% CIs for presenting with metastatic disease at diagnosis by race and ethnicity, stratified by sarcoma category and age group strata (crude results are given in eTable 5 in the Supplement). In the pediatric age group, we did not observe significant racial disparities in the odds of presenting with metastases at diagnosis for any of the subtypes evaluated. Conversely, in adults, we observed non-Hispanic Black patients to have an elevated odds of metastatic Ewing sarcoma of bone (OR, 5.00; 99% CI, 1.16-30.54), leiomyosarcoma (OR, 1.79; 99% CI, 1.42-2.24), and unclassified sarcomas (OR, 1.58; 99% CI, 1.04-2.37) compared with non-Hispanic White patients. The odds of metastatic leiomyosarcoma was also elevated in Hispanic (OR, 1.31; 99% CI, 1.04-1.65) and AIAN or API (OR, 1.45; 99% CI, 1.09-1.91) adults compared with non-Hispanic White adults. In the older age group strata, non-Hispanic Black patients had higher odds of metastatic leiomyosarcoma (OR, 1.59; 99% CI, 1.11-2.27) than non-Hispanic White patients, similar to the results observed in adults.

### Insurance Status and Metastasis at Diagnosis

The Table presents results from the analysis of insurance status on metastatic disease at diagnosis in the adult age group strata (crude results are presented in eTable 6 in the Supplement). In total, 12 890 adults with sarcoma diagnosed after 2007 were identified, and 322 (2.5%) were excluded from analysis for having missing insurance data. Across most sarcoma subtypes evaluated (6 of 8 subtypes), non-Medicaid insured patients had a lower odds of having metastases at diagnosis compared with Medicaid insured or uninsured patients; Ewing sarcoma of bone and osteosarcoma were the only subtypes evaluated to show null associations with both Medicaid insured and uninsured. In addition, insurance status appeared to attenuate the association observed with small-area SES among adult patients with liposarcoma (OR, 0.94; 99% CI, 0.80-1.10). However, non-Hispanic Black patients still had elevated odds of metastatic leiomyosarcoma (OR, 1.87; 99% CI, 1.41-2.48) and unclassified sarcomas (OR, 1.65; 99% CI, 1.01-2.67) compared with non-Hispanic White patients. The odds of non-Hispanic Black patients having metastatic Ewing sarcoma of bone also remained elevated but was extremely imprecise (OR, 9.15; 99% CI, 1.32-146.12). After adjustment for insurance status, there was no association of metastatic leiomyosarcoma with AIAN or API or Hispanic ethnicity.

**Table.** Multivariable Adjusted Odds Ratios for Metastases at Diagnosis in Adults Aged 20-65 y at Diagnosis, by Insurance Status, Stratified by Sarcoma Subtype: Surveillance, Epidemiology, and End Results 16 Registries, 2007-2015

Subtype	Patients, No.	Adjusted OR (99% CI)^a^					
		Insurance status^b^		Ordinal socioeconomic status	Race/ethnicity^c^		
		Medicaid	Uninsured		Non-Hispanic Black	Hispanic	AIAN or API
Osteosarcoma	677	1.68 (0.9-3.09)	1.04 (0.33-2.85)	0.94 (0.77-1.15)	1.3 (0.58-2.79)	1.11 (0.55-2.18)	0.85 (0.29-2.13)
Ewing sarcoma of bone	255	1.6 (0.63-4.06)	0.99 (0.14-5.79)	1.17 (0.89-1.56)	9.15 (1.32-146.12)	1.48 (0.57-3.81)	0.67 (0.08-3.85)
Synovial sarcoma	777	2.15 (1.11-4.09)	2.04 (0.86-4.61)	0.98 (0.81-1.18)	0.91 (0.36-2.1)	0.91 (0.49-1.68)	1.37 (0.58-3.03)
Gastrointestinal stromal tumor	2350	1.71 (1.18-2.44)	1.72 (1.08-2.71)	0.97 (0.89-1.07)	0.84 (0.58-1.21)	0.82 (0.56-1.2)	0.86 (0.58-1.26)
Leiomyosarcoma	3403	1.46 (1.1-1.95)	0.94 (0.59-1.45)	0.96 (0.89-1.04)	1.87 (1.41-2.48)	1.23 (0.92-1.63)	1.31 (0.92-1.85)
Liposarcoma	2957	2.27 (1.33-3.78)	2.44 (1.06-5.04)	0.94 (0.8-1.1)	0.79 (0.35-1.58)	0.78 (0.44-1.33)	1.31 (0.67-2.41)
Vascular tumors	564	1.9 (1.06-3.44)	1.13 (0.34-3.56)	0.95 (0.8-1.14)	0.79 (0.36-1.69)	1.08 (0.56-2.08)	0.91 (0.4-1.98)
Unclassified sarcomas	1585	1.71 (1.1-2.63)	1.32 (0.69-2.4)	0.92 (0.81-1.04)	1.65 (1.01-2.67)	1.34 (0.85-2.09)	1.22 (0.67-2.13)

Abbreviations: AIAN, American Indian and Alaska Native; API, Asian Pacific Islander; OR, odds ratio.

^a^ Adjusted for insurance status, area-level socioeconomic status (ordinal), race, sex, age at diagnosis, and year of diagnosis.

^b^ Reference category = insured.

^c^ Reference category = non-Hispanic white.

## Discussion

To our knowledge, this is the largest analysis aimed at ascertaining whether SES and race/ethnicity were independently associated with metastatic presentation of sarcoma. Overall, we found no association with small-area SES across most of the 25 sarcoma subtype–age group combinations evaluated. However, compared with having non-Medicaid insurance, having Medicaid or no insurance in adults was associated with an increased odds of metastases at diagnosis in 6 of the 8 subtypes evaluated; osteosarcoma and Ewing sarcoma were the only 2 subtypes not associated with insurance status. In addition, we observed an increased odds of presenting with metastases in non-Hispanic Black adults diagnosed with leiomyosarcoma and unclassified sarcomas that was independent of SES and insurance status. Our results suggest that those without insurance or who are underinsured are more likely to present with metastatic disease at diagnosis for several STS subtypes. Also, the fact that SES-related factors were not associated with the odds of metastatic presentation in patients with osteosarcoma or Ewing sarcoma, or with the racial disparities observed with metastatic leiomyosarcoma or unclassified sarcomas, indicates that factors other than diagnostic delay may contribute to the metastatic progression of these subtypes.

A limitation of our analysis is that we used area-level SES measured at the Census tract level as an indicator of individual-level SES. Although Census tracts comprise homogeneous populations,^32^ the variation in SES measures among individuals within a Census tract^33,34^ and the independent effects of individual and area level SES on health-related conditions^35,36,37^ indicate that area-based measures of SES should not be interpreted as if they were collected from individuals.^38^ Rather, area-based measures capture information on community and neighborhood factors that influence health, which are generally conceptualized to be independent of individual SES characteristics.^38^ We, therefore, sought to further explore the influence of SES on metastatic disease at presentation by evaluating insurance status, one of the few measures of SES captured by SEER at the individual level.

Across most STS subtypes evaluated in the adult age group strata, having Medicaid or no insurance was associated with an increased risk of presenting with metastases compared with having private insurance. Both Medicaid insured and uninsured patients can experience numerous barriers to receiving medical care, including reduced access to regular sources of health care services, prolonged referral times to specialists, an inability to navigate the health care system, transportation issues, or poor psychosocial support.^39,40,41,42,43,44^ Our study concurs with a prior analysis^45^ that reported an elevated risk of metastatic presentation in Medicaid insured patients diagnosed with STS of the extremities. Moreover, it builds on prior work in part because our analyses were stratified by sarcoma subtype, thereby providing a more detailed overview regarding the extent to which delayed access to medical care is associated with sarcoma stage at presentation.

Notably, we observed that insurance status and small-area SES were not associated with metastases at diagnosis of osteosarcoma or Ewing sarcoma, the 2 bone sarcoma subtypes evaluated in adults. These results both contrast with and confirm prior analyses. For instance, the finding that insurance status is not associated with metastatic osteosarcoma concurs with reports of a genetic predisposition to metastatic disease^46,47,48^ and null associations with SES measured at the county level,^2^ but is in contrast with our prior observation of a higher prevalence of metastases in countries with lower human development indices,^49^ which may harbor greater barriers in providing timely access to medical care. One possible explanation for these discrepant findings is that the population of patients with metastatic osteosarcoma is composed of both individuals with genetic susceptibility and those who experienced diagnostic delays, although the attributable proportion of each remains unexamined. Similarly, Ewing sarcoma tumors were not associated with either small-area SES or insurance status in adults, a finding consistent with prior reports.^3^ Future genetic analyses should be pursued to identify whether there exist risk variants for metastatic Ewing sarcoma.

We observed non-Hispanic Black adults to have a higher odds of metastatic leiomyosarcoma and unclassified sarcomas at diagnosis than non-Hispanic White adults after controlling for SES and insurance status. It is unclear what factors may underlie these observations. That they were independent of SES-related factors in our analysis highlights the possibility that genetic variants associated with ancestry may contribute to an inherently aggressive tumor phenotype, as hypothesized with other cancer types.^50,51,52^ However, we note that a single measure of SES cannot fully characterize the complexities and multifactorial nature of racial disparities in health. Other social, cultural, or lifestyle factors that are related to access to medical care, but that are unaccounted for by use of small-area SES or insurance status, may have confounded our observations. Further research is needed to determine whether there are associations between genetic ancestry and stage at diagnosis of these subtypes.

### Limitations

In addition to the use of an area-level SES measure, there are several additional limitations with our analyses that should be considered. First, the Medicaid insured category likely includes uninsured patients who were found to qualify for public health insurance after their cancer diagnosis, at which point they were retroactively coded as having Medicaid.^53,54^ We speculate that this misclassification likely inflated associations with Medicaid insurance and may explain the seemingly counterintuitive finding that Medicaid insured patients had a similar or higher risk of metastasis than uninsured patients. We also note that small sample sizes may have resulted in null associations with the uninsured category in instances when Medicaid insured patients had an increased risk. Second, the SES measure used in our analysis is a composite of several SES characteristics, each of which may have an effect on health that differs in direction or magnitude. However, the individual SES characteristics were not available for analysis. Third, the quality of sarcoma pathology^55,56^ and the possible inaccuracy of *ICD-O-3* coding of sarcomas^57,58^ is a limitation of our study and sarcoma research in general. Fourth, we note that SEER race/ethnicity categories are reported to be in high agreement with self-report race, except for patients identified as AIAN,^59^ but are imperfect measures of genomic ancestry.^60^

## Conclusions

In the current study, we provide evidence that factors related to diagnostic delay, including having Medicaid insurance or no insurance, were associated with an increased risk of presenting with more advanced staged STS in adults, but other factors were more likely associated with metastases at diagnosis for osteosarcoma and Ewing sarcoma, as well as leiomyosarcoma and unclassified sarcomas in non-Hispanic Black adults. These data may be used to guide efforts to detect metastatic sarcoma earlier to improve patient outcomes.

## Notes

Supplement. **eTable 1.** Soft Tissue and Bone Sarcoma Classifications and *ICD-O-3* Histology Codes **eTable 2.** Multivariable Adjusted OR for Metastatic Sarcoma by SES, Stratified by Age Group and Sarcoma Subtype: SEER 16 Registries, 2001–2015 **eTable 3.** Distribution of Sarcoma Cases by Demographic and Clinical Characteristics: SEER 16 Registries, 2001–2015 **eTable 4.** Unadjusted OR for Metastatic Sarcoma by SES, Stratified by Age Group and Sarcoma Subtype SEER 16 Registries, 2001–2015 **eTable 5.** Unadjusted OR for Metastatic Sarcoma by Race/Ethnicity, Stratified by Age Group and Sarcoma Subtype: SEER 16 Registries, 2001–2015 **eTable 6.** Unadjusted OR for Metastases at Diagnosis in Adults (20–65 Years at Diagnosis) by Insurance Status, Stratified by Sarcoma Subtype: SEER 16 Registries (2007–2015)
