# Supplementary material for: Associations of Socioeconomic Status, Public vs Private Insurance, and Race/Ethnicity With Metastatic Sarcoma at Diagnosis
Source: JAMA Netw Open. 2020 Aug 7;3(8):e2011087. doi: 10.1001/jamanetworkopen.2020.11087 (PMC7414392; doi:10.1001/jamanetworkopen.2020.11087)
Supplement: Supplement. — eTable 1. Soft Tissue and Bone Sarcoma Classifications and ICD-O-3 Histology Codes eTable 2. Multivariable Adjusted OR for Metastatic Sarcoma by SES, Stratified by Age Group and Sarcoma Subtype: SEER 16 Registries, 2001–2015 eTable 3. Distribution of Sarcoma Cases by Demographic and Clinical Characteristics: SEER 16 Registries, 2001–2015 eTable 4. Unadjusted OR for Metastatic Sarcoma by SES, Stratified by Age Group and Sarcoma Subtype SEER 16 Registries, 2001–2015 eTable 5. Unadjusted OR for Metastatic Sarcoma by Race/Ethnicity, Stratified by Age Group and Sarcoma Subtype: SEER 16 Registries, 2001–2015 eTable 6. Unadjusted OR for Metastases at Diagnosis in Adults (20–65 Years at Diagnosis) by Insurance Status, Stratified by Sarcoma Subtype: SEER 16 Registries (2007–2015) [file jamanetwopen-3-e2011087-s001.pdf]

Supplementary Online Content

Diessner BJ, Weigel BJ, Murugan P, Zhang L, Poynter JN, Spector LG. Associations of socioeconomic status, public vs private insurance, and race/ethnicity with metastatic sarcoma at diagnosis. *JAMA Netw Open*. 2020;3(8):e2011087. doi:10.1001/jamanetworkopen.2020.11087

eTable 1. Soft Tissue and Bone Sarcoma Classifications and *ICD-O-3* Histology Codes

eTable 2. Multivariable Adjusted OR for Metastatic Sarcoma by SES, Stratified by Age Group and Sarcoma Subtype: SEER 16 Registries, 2001–2015

eTable 3. Distribution of Sarcoma Cases by Demographic and Clinical Characteristics: SEER 16 Registries, 2001-2015

eTable 4. Unadjusted OR for Metastatic Sarcoma by SES, Stratified by Age Group and Sarcoma Subtype SEER 16 Registries, 2001-2015

eTable 5. Unadjusted OR for Metastatic Sarcoma by Race/Ethnicity, Stratified by Age Group and Sarcoma Subtype: SEER 16 Registries, 2001–2015

eTable 6. Unadjusted OR for Metastases at Diagnosis in Adults (20–65 Years at Diagnosis) by Insurance Status, Stratified by Sarcoma Subtype: SEER 16 Registries (2007–2015)

This supplementary material has been provided by the authors to give readers additional information about their work.

© 2020 Diessner BJ et al. *JAMA Network Open.*

eTable 1. Soft Tissue and Bone Sarcoma Classifications and *ICD-O-3* Histology Codes	
**Histologic classifications**	**ICD-O-3 histology codes**
**Soft tissue sarcomas***	
Liposarcoma	8850, 8851-8855, 8858
Fibroblastic/Myofibroblastic tumors	8810, 8811, 8815, 8815, 8825, 8832, 8840
Leiomyosarcoma	8890
Malignant glomus tumor	8711
Embryonal rhabdomyosarcoma	8910
Alveolar rhabdomyosarcoma	8920
Other rhabdomyosarcoma	8900, 8901, 8912
Malignant vascular tumors	9120, 9133
Synovial sarcoma	9040, 9041,9043
Epithelioid sarcoma	8804
Alveolar soft part sarcoma	9581
Clear Cell Sarcoma of Soft tissue	9044
Extraskeletal mesenchymal chondrosarcoma	9240
Malignant ossifying fibromyxoid tumor	8842
Myoepithelial carcinoma	8982
Malignant phosphaturic mesenchymal tumor	8990
Extra skeletal myxoid chondrosarcoma	9231
Ewing sarcoma soft tissue	9260, 9364
Desmoplastic small round cell tumor	8806
Extra-renal rhabdoid tumor	8963
Gastrointestinal stromal tumor	8936
Malignant peripheral nerve sheath tumor	8921, 9540, 9561, 9571, 9580
Extra-skeletal osteosarcoma	9180
Undifferentiated pleomorphic sarcoma	8830
unclassified sarcomas	8800 – 8803, 8805
**Bone Sarcomas****	
Osteosarcoma	9180 – 9183, 9185, 9187, 9192 - 9194
Chondrosarcoma	9220, 9221, 9240, 9242, 9243
Fibrosarcoma of bone	8810
Malignancy in giant cell tumor of bone	9250
Ewing sarcoma of bone	9364, 9260
Undifferentiated pleomorphic sarcoma of bone	8830
Chordoma	9370
Epithelioid haemangioendothelioma of bone	9133
Angiosarcoma of bone	9120
Leiomyosarcoma of bone	8890
Liposarcoma of bone	8850
*Occurring in any sites other than bones and joints. **Occurring in bones and joints. Only ICD-O-3 codes with malignant behavior included. Only subtypes with > 100 metastatic cases within an age group strata were evaluated.	

© 2020 Diessner BJ et al. *JAMA Network Open.*

eTable 2. Multivariable Adjusted OR for Metastatic Sarcoma by SES, Stratified by Age Group and Sarcoma Subtype: SEER 16 Registries, 2001–2015[a]						
	Small-area SES					
**Pediatric (< 20 years)**	Q1	Q2	Q3	Q4	Q5	Ordinal SES[b]
Osteosarcoma	Ref	0.97 (0.6, 1.56)	0.75 (0.45, 1.25)	0.76 (0.45, 1.28)	0.66 (0.38, 1.14)	0.9 (0.79, 1.01)
ES of bone	Ref	0.63 (0.33, 1.21)	0.5 (0.26, 0.93)	0.69 (0.36, 1.35)	0.64 (0.34, 1.21)	0.94 (0.81, 1.08)
ARMS	Ref	0.89 (0.41, 1.95)	0.84 (0.37, 1.93)	0.65 (0.27, 1.52)	0.77 (0.34, 1.76)	0.92 (0.77, 1.11)
Embryonal RMS	Ref	0.94 (0.45, 1.96)	0.76 (0.35, 1.65)	1.02 (0.48, 2.16)	0.67 (0.31, 1.44)	0.93 (0.78, 1.1)
**Adult (20 - 65 years)**						
Osteosarcoma	Ref	0.76 (0.4, 1.42)	1.1 (0.61, 2)	0.87 (0.46, 1.63)	0.7 (0.36, 1.33)	0.94 (0.81, 1.09)
ES of bone	Ref	1.08 (0.45, 2.62)	1.1 (0.45, 2.71)	1.27 (0.51, 3.16)	1.01 (0.42, 2.5)	1.01 (0.83, 1.23)
Chondrosarcoma	Ref	1.93 (0.83, 4.79)	1.61 (0.69, 4.02)	1.52 (0.66, 3.76)	1.52 (0.66, 3.8)	1.03 (0.87, 1.23)
Other RMS	Ref	1.62 (0.62, 4.32)	1.31 (0.51, 3.4)	1.06 (0.4, 2.82)	1.28 (0.48, 3.46)	1 (0.81, 1.25)
F/MF tumors	Ref	0.7 (0.32, 1.48)	0.96 (0.49, 1.91)	0.82 (0.41, 1.68)	0.71 (0.35, 1.45)	0.94 (0.81, 1.11)
Synovial sarcoma	Ref	0.75 (0.42, 1.34)	0.6 (0.32, 1.11)	0.57 (0.3, 1.04)	0.71 (0.39, 1.3)	0.91 (0.79, 1.05)
ES of soft tissue	Ref	1.2 (0.5, 2.91)	1.61 (0.68, 3.9)	0.66 (0.25, 1.72)	0.93 (0.37, 2.37)	0.93 (0.76, 1.14)
MPNST	Ref	1 (0.48, 2.07)	0.95 (0.44, 2)	1.51 (0.75, 3.09)	0.55 (0.22, 1.32)	0.96 (0.8, 1.15)
DSRCT	Ref	1.27 (0.28, 5.78)	0.84 (0.19, 3.56)	0.48 (0.1, 2.17)	0.55 (0.12, 2.34)	0.81 (0.57, 1.13)
GIST	Ref	1.04 (0.75, 1.45)	0.91 (0.65, 1.26)	0.88 (0.63, 1.22)	0.9 (0.65, 1.25)	0.96 (0.89, 1.04)
Leiomyosarcoma	Ref	1.05 (0.8, 1.36)	0.84 (0.64, 1.1)	0.94 (0.72, 1.22)	0.86 (0.66, 1.13)	0.96 (0.9, 1.02)
Liposarcoma	Ref	0.92 (0.55, 1.54)	0.92 (0.56, 1.52)	0.71 (0.42, 1.21)	0.52 (0.3, 0.9)	0.85 (0.76, 0.96)
Malignant vascular tumors	Ref	1.05 (0.57, 1.93)	0.8 (0.43, 1.47)	0.9 (0.49, 1.66)	0.73 (0.39, 1.36)	0.92 (0.81, 1.06)
UPS	Ref	0.92 (0.43, 1.95)	0.7 (0.32, 1.49)	0.96 (0.48, 1.97)	0.77 (0.38, 1.6)	0.96 (0.81, 1.13)
Unclassified sarcomas	Ref	0.7 (0.45, 1.09)	0.74 (0.48, 1.13)	0.64 (0.41, 1)	0.64 (0.41, 1)	0.91 (0.82, 1)
**Older Adult (65 years +)**						
GIST	Ref	1.09 (0.75, 1.57)	0.96 (0.65, 1.41)	1.02 (0.7, 1.48)	0.88 (0.6, 1.29)	0.97 (0.89, 1.05)
Leiomyosarcoma	Ref	1.08 (0.73, 1.59)	1.06 (0.72, 1.56)	1.11 (0.75, 1.64)	0.93 (0.63, 1.37)	0.98 (0.9, 1.07)
Liposarcoma	Ref	1.06 (0.53, 2.18)	1.21 (0.63, 2.41)	0.94 (0.48, 1.89)	1.23 (0.65, 2.42)	1.03 (0.9, 1.19)
Malignant vascular tumors	Ref	0.88 (0.41, 1.91)	1.05 (0.51, 2.19)	0.92 (0.46, 1.89)	1.14 (0.57, 2.36)	1.04 (0.89, 1.21)
UPS	Ref	0.57 (0.27, 1.17)	0.71 (0.36, 1.4)	0.59 (0.3, 1.19)	0.59 (0.3, 1.15)	0.91 (0.78, 1.06)
Unclassified sarcomas	Ref	0.6 (0.35, 1.02)	0.6 (0.36, 1.01)	0.62 (0.37, 1.03)	0.58 (0.35, 0.96)	0.91 (0.81, 1.02)

Abbreviations: ES = Ewing sarcoma; RMS = rhabdomyosarcoma; ARMS = alveolar RMS; F/MF. = fibroblastic or myofibroblastic; MPNST = malignant peripheral nerve sheath tumor; DSRCT = desmoplastic small round cell tumor, GIST = gastrointestinal stromal tumor, UPS = undifferentiated pleomorphic sarcoma; Ref = reference category; Q1 corresponds to lowest SES quintile, Q5 corresponds to highest. [a]All data reported as odds ratio (99% CI) and are adjusted for race, sex, age at diagnosis, and year of diagnosis. [b] Results obtained from evaluating SES as an ordinal variable.

© 2020 Diessner BJ et al. *JAMA Network Open.*

eTable 3. Distribution of Sarcoma Cases by Demographic and Clinical Characteristics: SEER 16 Registries, 2001-2015[a]

	Total, No.	No. (%)												
		Sex		Race/ethnicity				SES					Stage	
		Male	Female	NHW	NHB	H	AIAN/API	Q1	Q2	Q3	Q4	Q5	L/R	D
**All ages at diagnosis**														
Bone sarcomas	5616	3160 (56.3)	2456 (43.7)	3368 (60)	521 (9.3)	1324 (23.6)	403 (7.2)	993 (17.7)	1071 (19.1)	1145 (20.4)	1125 (20)	1282 (22.8)	4462 (79.5)	1154 (20.5)
Soft tissue sarcomas	41,721	21,183 (50.8)	20,538 (49.2)	26,607 (63.8)	5152 (12.3)	6180 (14.8)	3782 (9.1)	6860 (16.4)	7605 (18.2)	8298 (19.9)	9067 (21.7)	9891 (23.7)	34,493 (82.7)	7228 (17.3)
**Pediatric (<20 y)**														
Osteosarcoma	1521	854 (56.1)	667 (43.9)	678 (44.6)	229 (15.1)	490 (32.2)	124 (8.2)	302 (19.9)	329 (21.6)	297 (19.5)	296 (19.5)	297 (19.5)	1176 (77.3)	345 (22.7)
ES of bone	893	539 (60.4)	354 (39.6)	587 (65.7)	21 (2.4)	221 (24.7)	64 (7.2)	127 (14.2)	167 (18.7)	210 (23.5)	159 (17.8)	230 (25.8)	612 (68.5)	281 (31.5)
ARMS	474	254 (53.6)	220 (46.4)	213 (44.9)	77 (16.2)	135 (28.5)	49 (10.3)	88 (18.6)	104 (21.9)	85 (17.9)	90 (19)	107 (22.6)	251 (53)	223 (47)
Embryonal RMS	725	447 (61.7)	278 (38.3)	398 (54.9)	104 (14.3)	180 (24.8)	43 (5.9)	132 (18.2)	144 (19.9)	138 (19)	136 (18.8)	175 (24.1)	570 (78.6)	155 (21.4)
**Adult (20-65 y)**														
Osteosarcoma	1150	632 (55)	518 (45)	628 (54.6)	152 (13.2)	265 (23)	105 (9.1)	246 (21.4)	203 (17.7)	238 (20.7)	220 (19.1)	243 (21.1)	933 (81.1)	217 (18.9)
ES of bone	419	273 (65.2)	146 (34.8)	309 (73.7)	15 (3.6)	75 (17.9)	20 (4.8)	74 (17.7)	85 (20.3)	78 (18.6)	82 (19.6)	100 (23.9)	249 (59.4)	170 (40.6)
Chondrosarcoma	1633	862 (52.8)	771 (47.2)	1166 (71.4)	104 (6.4)	273 (16.7)	90 (5.5)	244 (14.9)	287 (17.6)	322 (19.7)	368 (22.5)	412 (25.2)	1492 (91.4)	141 (8.6)
Other RMS	342	198 (57.9)	144 (42.1)	176 (51.5)	57 (16.7)	73 (21.3)	36 (10.5)	64 (18.7)	62 (18.1)	74 (21.6)	71 (20.8)	71 (20.8)	212 (62)	130 (38)
F/MF tumors	5198	2480 (47.7)	2718 (52.3)	2960 (56.9)	980 (18.9)	786 (15.1)	472 (9.1)	906 (17.4)	922 (17.7)	1052 (20.2)	1083 (20.8)	1235 (23.8)	5051 (97.2)	147 (2.8)
Synovial sarcoma	1299	696 (53.6)	603 (46.4)	731 (56.3)	131 (10.1)	335 (25.8)	102 (7.9)	241 (18.6)	262 (20.2)	257 (19.8)	270 (20.8)	269 (20.7)	1072 (82.5)	227 (17.5)
ES of soft tissue	418	220 (52.6)	198 (47.4)	259 (62)	17 (4.1)	91 (21.8)	51 (12.2)	72 (17.2)	89 (21.3)	92 (22)	83 (19.9)	82 (19.6)	271 (64.8)	147 (35.2)

© 2020 Diessner BJ et al. *JAMA Network Open.*

MPNST	976	532 (54.5)	444 (45.5)	558 (57.2)	151 (15.5)	178 (18.2)	89 (9.1)	218 (22.3)	190 (19.5)	198 (20.3)	190 (19.5)	180 (18.4)	833 (85.3)	143 (14.7)
DSRCT	187	154 (82.4)	33 (17.6)	96 (51.3)	38 (20.3)	36 (19.3)	17 (9.1)	34 (18.2)	39 (20.9)	45 (24.1)	29 (15.5)	40 (21.4)	53 (28.3)	134 (71.7)
GIST	3647	2029 (55.6)	1618 (44.4)	1971 (54)	662 (18.2)	524 (14.4)	490 (13.4)	671 (18.4)	664 (18.2)	712 (19.5)	770 (21.1)	830 (22.8)	2730 (74.9)	917 (25.1)
Leiomyosarcoma	5689	1520 (26.7)	4169 (73.3)	3335 (58.6)	877 (15.4)	972 (17.1)	505 (8.9)	963 (16.9)	1043 (18.3)	1087 (19.1)	1316 (23.1)	1280 (22.5)	4240 (74.5)	1449 (25.5)
Liposarcoma	4620	2769 (59.9)	1851 (40.1)	2899 (62.7)	418 (9)	904 (19.6)	399 (8.6)	720 (15.6)	811 (17.6)	935 (20.2)	1007 (21.8)	1147 (24.8)	4343 (94)	277 (6)
Malignant vascular Tumors	877	463 (52.8)	414 (47.2)	567 (64.7)	96 (10.9)	135 (15.4)	79 (9)	137 (15.6)	172 (19.6)	181 (20.6)	191 (21.8)	196 (22.3)	550 (62.7)	327 (37.3)
UPS	1481	909 (61.4)	572 (38.6)	1026 (69.3)	134 (9)	225 (15.2)	96 (6.5)	240 (16.2)	237 (16)	288 (19.4)	330 (22.3)	386 (26.1)	1327 (89.6)	154 (10.4)
Unclassified sarcomas	2103	1125 (53.5)	978 (46.5)	1283 (61)	272 (12.9)	366 (17.4)	182 (8.7)	386 (18.4)	388 (18.4)	427 (20.3)	428 (20.4)	474 (22.5)	1637 (77.8)	466 (22.2)
**Older adult (>65 y)**														
GIST	2982	1457 (48.9)	1525 (51.1)	1840 (61.7)	439 (14.7)	281 (9.4)	422 (14.2)	504 (16.9)	571 (19.1)	556 (18.6)	656 (22)	695 (23.3)	2284 (76.6)	698 (23.4)
Leiomyosarcoma	2959	1186 (40.1)	1773 (59.9)	2202 (74.4)	311 (10.5)	262 (8.9)	184 (6.2)	468 (15.8)	553 (18.7)	604 (20.4)	612 (20.7)	722 (24.4)	2271 (76.7)	688 (23.3)
Liposarcoma	2620	1624 (62)	996 (38)	1933 (73.8)	143 (5.5)	317 (12.1)	227 (8.7)	361 (13.8)	455 (17.4)	515 (19.7)	605 (23.1)	684 (26.1)	2418 (92.3)	202 (7.7)
Malignant vascular Tumors	910	520 (57.1)	390 (42.9)	689 (75.7)	40 (4.4)	84 (9.2)	97 (10.7)	125 (13.7)	147 (16.2)	188 (20.7)	236 (25.9)	214 (23.5)	698 (76.7)	212 (23.3)
UPS	2319	1551 (66.9)	768 (33.1)	1959 (84.5)	84 (3.6)	153 (6.6)	123 (5.3)	285 (12.3)	414 (17.9)	487 (21)	517 (22.3)	616 (26.6)	2151 (92.8)	168 (7.2)
Unclassified sarcomas	1895	1049 (55.4)	846 (44.6)	1512 (79.8)	121 (6.4)	143 (7.5)	119 (6.3)	245 (12.9)	338 (17.8)	377 (19.9)	447 (23.6)	488 (25.8)	1531 (80.8)	364 (19.2)

Q1 corresponds to lowest SES quintile, Q5 corresponds to highest.
Abbreviations: AIAIN/API, American Indian, Alaskan native, Asian Pacific Islander; ARMS, alveolar rhabdomyosarcoma; B, Black; D, distant; DSRCT, desmoplastic small round cell tumor, GIST, gastrointestinal stromal tumor; ES, Ewing sarcoma; F/MF, fibroblastic or myofibroblastic; H, Hispanic; L/R, localized or regional; NH, Non-Hispanic; RMS, rhabdomyosarcoma; SEER, Surveillance, Epidemiology, and End Results Program; SES, socioeconomic status; UPS, undifferentiated pleomorphic sarcoma; W, White.

© 2020 Diessner BJ et al. *JAMA Network Open.*

[a]All data reported as number (percent of total).

eTable 4. Unadjusted OR for Metastatic Sarcoma by SES, Stratified by Age Group and Sarcoma Subtype SEER 16 Registries, 2001-2015[a]						
	Small-area SES					
**Pediatric (< 20 years)**	Q1	Q2	Q3	Q4	Q5	Ordinal SES[b]
Osteosarcoma	Ref	0.95 (0.6, 1.52)	0.72 (0.44, 1.18)	0.72 (0.44, 1.19)	0.62 (0.37, 1.03)	0.88 (0.79, 0.99)
ES of bone	Ref	0.63 (0.33, 1.19)	0.48 (0.26, 0.89)	0.64 (0.34, 1.21)	0.64 (0.35, 1.15)	0.93 (0.81, 1.06)
ARMS	Ref	1 (0.47, 2.11)	0.89 (0.4, 1.95)	0.7 (0.32, 1.52)	0.88 (0.42, 1.84)	0.94 (0.8, 1.11)
Embryonal RMS	Ref	0.93 (0.45, 1.94)	0.76 (0.35, 1.63)	1 (0.48, 2.1)	0.65 (0.31, 1.35)	0.92 (0.78, 1.08)
**Adult (20 - 65 years)**						
Osteosarcoma	Ref	0.74 (0.39, 1.37)	0.97 (0.55, 1.71)	0.79 (0.43, 1.44)	0.64 (0.34, 1.17)	0.92 (0.8, 1.05)
ES of bone	Ref	0.92 (0.4, 2.13)	0.91 (0.39, 2.15)	1.09 (0.47, 2.52)	0.85 (0.38, 1.91)	0.98 (0.82, 1.17)
Chondrosarcoma	Ref	1.78 (0.78, 4.35)	1.51 (0.66, 3.7)	1.45 (0.65, 3.52)	1.42 (0.64, 3.4)	1.02 (0.87, 1.21)
Other RMS	Ref	1.57 (0.61, 4.1)	1.23 (0.49, 3.11)	0.97 (0.38, 2.51)	1.17 (0.46, 2.99)	0.98 (0.8, 1.21)
F/MF tumors	Ref	0.7 (0.33, 1.48)	0.98 (0.5, 1.93)	0.89 (0.45, 1.77)	0.83 (0.43, 1.63)	0.98 (0.84, 1.14)
Synovial sarcoma	Ref	0.76 (0.42, 1.34)	0.62 (0.34, 1.12)	0.58 (0.32, 1.06)	0.75 (0.42, 1.33)	0.92 (0.8, 1.05)
ES of soft tissue	Ref	1.09 (0.47, 2.57)	1.36 (0.59, 3.17)	0.6 (0.24, 1.48)	0.82 (0.34, 1.99)	0.9 (0.74, 1.1)
MPNST	Ref	0.97 (0.47, 1.99)	0.92 (0.45, 1.89)	1.45 (0.74, 2.86)	0.51 (0.21, 1.16)	0.95 (0.8, 1.11)
DSRCT	Ref	1.4 (0.33, 6.09)	1.11 (0.28, 4.29)	0.59 (0.14, 2.4)	0.67 (0.17, 2.46)	0.84 (0.62, 1.14)
GIST	Ref	1.07 (0.78, 1.48)	0.95 (0.69, 1.3)	0.92 (0.67, 1.27)	0.95 (0.7, 1.3)	0.98 (0.91, 1.05)
Leiomyosarcoma	Ref	0.95 (0.73, 1.22)	0.74 (0.57, 0.95)	0.81 (0.64, 1.04)	0.7 (0.55, 0.9)	0.92 (0.87, 0.97)
Liposarcoma	Ref	0.91 (0.55, 1.53)	0.92 (0.56, 1.52)	0.71 (0.43, 1.19)	0.53 (0.31, 0.89)	0.86 (0.77, 0.96)
Malignant vascular tumors	Ref	1.07 (0.59, 1.96)	0.8 (0.44, 1.46)	0.92 (0.51, 1.67)	0.72 (0.4, 1.31)	0.92 (0.81, 1.05)
UPS	Ref	0.9 (0.42, 1.88)	0.69 (0.33, 1.45)	0.95 (0.48, 1.89)	0.75 (0.38, 1.5)	0.95 (0.82, 1.11)
Unclassified sarcomas	Ref	0.66 (0.43, 1.01)	0.68 (0.45, 1.03)	0.6 (0.39, 0.91)	0.57 (0.38, 0.87)	0.88 (0.8, 0.97)
**Older Adult (65 years +)**						
GIST	Ref	1.06 (0.74, 1.53)	0.93 (0.64, 1.35)	0.96 (0.67, 1.37)	0.82 (0.58, 1.18)	0.95 (0.88, 1.03)
Leiomyosarcoma	Ref	0.96 (0.66, 1.4)	0.94 (0.65, 1.36)	0.95 (0.66, 1.38)	0.8 (0.55, 1.15)	0.95 (0.88, 1.03)
Liposarcoma	Ref	1.04 (0.52, 2.12)	1.2 (0.63, 2.39)	0.91 (0.47, 1.82)	1.19 (0.65, 2.31)	1.02 (0.89, 1.18)
Malignant vascular tumors	Ref	0.88 (0.42, 1.87)	1 (0.5, 2.02)	0.89 (0.46, 1.78)	1.04 (0.53, 2.08)	1.01 (0.87, 1.18)
UPS	Ref	0.49 (0.24, 1)	0.61 (0.32, 1.16)	0.5 (0.26, 0.98)	0.49 (0.26, 0.93)	0.87 (0.75, 1.01)
Unclassified sarcomas	Ref	0.54 (0.32, 0.9)	0.56 (0.34, 0.92)	0.55 (0.34, 0.9)	0.52 (0.32, 0.84)	0.89 (0.8, 0.99)

Abbreviations: ES = Ewing sarcoma; RMS = rhabdomyosarcoma; ARMS = Alveolar RMS; F/MF. = Fibroblastic or Myofibroblastic; MPNST = malignant peripheral nerve sheath tumor; DSRCT = Desmoplastic small round cell tumor, GIST = gastrointestinal stromal tumor, UPS = undifferentiated pleomorphic sarcoma; Ref = reference category; Q1 corresponds to lowest SES quintile, Q5 corresponds to highest. [a]All data reported as odds ratio (99% CI). [b] Results obtained from evaluating SES as an ordinal variable.

© 2020 Diessner BJ et al. *JAMA Network Open.*

eTable 5. Unadjusted OR for Metastatic Sarcoma by Race/Ethnicity, Stratified by Age Group and Sarcoma Subtype: SEER 16 Registries, 2001–2015[a]

	Race/Ethnicity			
**Pediatric (< 20 years)**	NHW	NHB	Hispanic	AIAN/API
Osteosarcoma	Ref	1.2 (0.74, 1.9)	1.36 (0.94, 1.95)	0.98 (0.51, 1.79)
ES of bone	Ref	0.74 (0.16, 2.54)	1.39 (0.9, 2.12)	0.99 (0.46, 2.04)
ARMS	Ref	1.03 (0.52, 2.05)	1.03 (0.58, 1.81)	1.33 (0.58, 3.04)
Embryonal RMS	Ref	1.07 (0.51, 2.09)	1.29 (0.73, 2.22)	1.05 (0.34, 2.73)
**Adult (20 - 65 years)**				
Osteosarcoma	Ref	1.47 (0.82, 2.56)	1.32 (0.81, 2.11)	1.16 (0.55, 2.26)
ES of bone	Ref	4.51 (1.08, 26.98)	1.36 (0.69, 2.66)	1.09 (0.3, 3.64)
Chondrosarcoma	Ref	0.78 (0.23, 1.97)	1.18 (0.64, 2.08)	1.05 (0.34, 2.58)
Other RMS	Ref	0.93 (0.4, 2.07)	1.31 (0.63, 2.71)	0.45 (0.13, 1.29)
F/MF tumors	Ref	0.72 (0.37, 1.32)	1.04 (0.55, 1.85)	1.08 (0.49, 2.14)
Synovial sarcoma	Ref	1.01 (0.51, 1.88)	1.02 (0.64, 1.58)	1.02 (0.47, 2.01)
ES of soft tissue	Ref	0.92 (0.19, 3.5)	1.8 (0.94, 3.43)	1.42 (0.61, 3.18)
MPNST	Ref	1.18 (0.59, 2.22)	1.07 (0.55, 1.97)	1.37 (0.6, 2.88)
DSRCT	Ref	0.6 (0.21, 1.77)	1.06 (0.34, 3.62)	0.85 (0.2, 4.43)
GIST	Ref	0.92 (0.7, 1.19)	0.93 (0.69, 1.24)	0.99 (0.73, 1.33)
Leiomyosarcoma	Ref	2.02 (1.64, 2.5)	1.39 (1.12, 1.73)	1.53 (1.16, 2)
Liposarcoma	Ref	1.11 (0.61, 1.88)	1.19 (0.78, 1.75)	1.21 (0.67, 2.05)
Malignant vascular tumors	Ref	1.17 (0.65, 2.09)	1.2 (0.72, 1.98)	1.22 (0.64, 2.28)
UPS	Ref	1.24 (0.56, 2.49)	1.14 (0.6, 2.05)	1.31 (0.52, 2.86)
Unclassified sarcomas	Ref	1.7 (1.15, 2.5)	1.41 (0.98, 2.01)	1.15 (0.69, 1.86)
**Older Adult (65 years +)**				
GIST	Ref	1.01 (0.73, 1.38)	1.18 (0.8, 1.7)	0.69 (0.47, 0.97)
Leiomyosarcoma	Ref	1.89 (1.34, 2.64)	1.26 (0.85, 1.85)	1.6 (1.02, 2.45)
Liposarcoma	Ref	0.51 (0.14, 1.34)	1.22 (0.69, 2.05)	0.77 (0.34, 1.53)
Malignant vascular tumors	Ref	1.57 (0.59, 3.8)	1.46 (0.73, 2.79)	1.48 (0.78, 2.73)
UPS	Ref	2.86 (1.21, 6.01)	1.22 (0.5, 2.56)	1.84 (0.79, 3.76)
Unclassified sarcomas	Ref	1.56 (0.86, 2.7)	2.1 (1.26, 3.42)	1.32 (0.7, 2.35)

ES = Ewing sarcoma; RMS = rhabdomyosarcoma; ARMS = Alveolar RMS; F/MF. = fibroblastic or myofibroblastic; MPNST = malignant peripheral nerve sheath tumor; DSRCT = Desmoplastic small round cell tumor, GIST = gastrointestinal stromal tumor, UPS = undifferentiated pleomorphic sarcoma; Ref = reference. NH = Non-Hispanic; H = Hispanic; W = White; B = Black; AIAIN/API = American Indian, Alaskan native, Asian Pacific Islander. [a]All data reported as odds ratio (99% CI).

© 2020 Diessner BJ et al. *JAMA Network Open.*

eTable 6. Unadjusted OR for Metastases at Diagnosis in Adults (20–65 Years at Diagnosis) by Insurance Status, Stratified by Sarcoma Subtype: SEER 16 Registries (2007–2015)[a]

	Insurance status[b]		
subtype	Insured	Medicaid	Uninsured
Osteosarcoma	Ref	1.68 (0.95, 2.91)	1.19 (0.39, 3.06)
ES of bone	Ref	1.31 (0.6, 2.87)	1.19 (0.22, 5.68)
Synovial sarcoma	Ref	1.94 (1.07, 3.44)	1.99 (0.88, 4.19)
GIST	Ref	1.61 (1.14, 2.25)	1.71 (1.08, 2.66)
Leiomyosarcoma	Ref	1.66 (1.27, 2.15)	1.09 (0.71, 1.65)
Liposarcoma	Ref	2.22 (1.35, 3.54)	2.27 (1.01, 4.55)
Vascular tumors	Ref	2.06 (1.18, 3.63)	1.24 (0.39, 3.7)
Unclassified sarcomas	Ref	1.98 (1.32, 2.94)	1.47 (0.79, 2.6)

Abbreviations: ES = Ewing sarcoma; GIST = gastrointestinal stromal tumor; NHB = Non-Hispanic Black; AIAN/API = American Indian, Alaskan Native, Asian Pacific Islander; [a] All data reported as odds ratio (99% CI). [b] Reference category = insured;

© 2020 Diessner BJ et al. *JAMA Network Open.*
